# Artificial intelligence-enabled microfluidic cytometer using gravity-driven slug flow for rapid CD4^+^ T cell quantification in whole blood

**DOI:** 10.1038/s41378-025-00881-y

**Published:** 2025-02-28

**Authors:** Desh Deepak Dixit, Tyler P. Graf, Kevin J. McHugh, Peter B. Lillehoj

**Affiliations:** 1 Department of Mechanical Engineering, Rice University, Houston, TX USA; 2 Department of Bioengineering, Rice University, Houston, TX USA; 3 Department of Chemistry, Rice University, Houston, TX USA

**Keywords:** Engineering, Physics

## Abstract

The quantification of immune cell subpopulations in blood is important for the diagnosis, prognosis and management of various diseases and medical conditions. Flow cytometry is currently the gold standard technique for cell quantification; however, it is laborious, time-consuming and relies on bulky/expensive instrumentation, limiting its use to laboratories in high-resource settings. Microfluidic cytometers offering enhanced portability have been developed that are capable of rapid cell quantification; however, these platforms involve tedious sample preparation and processing protocols and/or require the use of specialized/expensive instrumentation for flow control and cell detection. Here, we report an artificial intelligence-enabled microfluidic cytometer for rapid CD4^+^ T cell quantification in whole blood requiring minimal sample preparation and instrumentation. CD4^+^ T cells in blood are labeled with anti-CD4 antibody-coated microbeads, which are driven through a microfluidic chip via gravity-driven slug flow, enabling pump-free operation. A video of the sample flowing in the chip is recorded using a microscope camera, which is analyzed using a convolutional neural network-based model that is trained to detect bead-labeled cells in the blood flow. The functionality of this platform was evaluated by analyzing fingerprick blood samples obtained from healthy donors, which revealed its ability to quantify CD4^+^ T cells with similar accuracy as flow cytometry (<10% deviation between both methods) while being at least 4× faster, less expensive, and simpler to operate. We envision that this platform can be readily modified to quantify other cell subpopulations in blood by using beads coated with different antibodies, making it a promising tool for performing cell count measurements outside of laboratories and in low-resource settings.

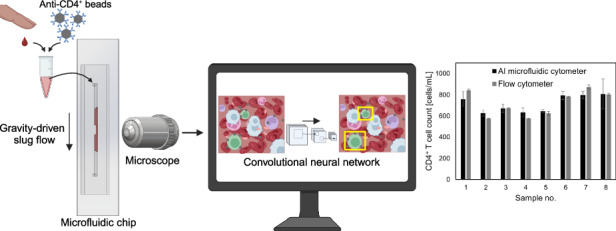

## Introduction

CD4^+^ T cells are a subset of thymus-derived lymphocytes that play an important role in mediating the immune response. Impaired immune function caused by some pathogenic infections, cancers, chemotherapeutic treatments, or immunosuppressive therapies results in a depletion of CD4^+^ T cell populations^1–3^. Thus, the CD4^+^ T cell count is a reliable indicator of immune status and serves as a useful diagnostic and prognostic marker for several diseases, including human immunodeficiency virus (HIV) infection and lymphomas as well as for monitoring treatment efficacy. Currently, the CD4^+^ T cell count is used by clinicians to monitor the progression of HIV infection and efficacy of antiretroviral therapy (ART) in HIV patients^4^, as well as diagnosing acquired immunodeficiency syndrome (AIDS)^5^. Decreased CD4^+^ T cell counts are also associated with an increased risk of non-AIDS defining cancers, including Kaposi’s sarcoma and non-Hodgkin lymphoma^6^, making it useful for cancer screening. Studies have also shown that CD4^+^ T cell levels strongly correlate with COVID-19 infection severity^7,8^, supporting its use as a prognostic test for COVID-19. Current methods for counting CD4^+^ T cells in blood rely on flow cytometry, which is the “gold standard” technique for cell quantification. While capable of highly accurate measurements, flow cytometry is time-consuming, requires bulky/expensive instrumentation, and involves laborious sample preparation and processing performed by highly trained operators, all of which limits its use to laboratories in high-resource settings^9^.

Microfluidic platforms for immune cell quantification have been developed which are faster, less expensive and offer enhanced portability compared with conventional flow cytometry. The vast majority of these devices employ antibodies that specifically bind to surface proteins expressed on the target cell(s), enabling them to be tagged to a bead, fluorophore or enzyme and subsequently detected via impedance^10–13^, electrochemical^14,15^ or optical/fluorescence^16,17^ measurements. While these platforms have demonstrated the ability to accurately quantify immune cells, they rely on specialized/expensive instrumentation for flow control (e.g., syringe pump) or cell detection (e.g., impedance analyzer, potentiostat, microplate reader, fluorescence microscope). Furthermore, many of these techniques involve lengthy (>1 h) and/or complicated sample preparation protocols (e.g., blood separation, red blood cell (RBC) lysis, cell labeling or cell immobilization)^18,19^.

Recently, artificial intelligence (AI) has made significant progress in image-recognition tasks, including in the assessment of medical images for the detection, characterization and monitoring of diseases^20^. Toward this end, machine learning (ML) algorithms have been employed for real-time cell identification and quantification in complex heterogeneous populations using static microscopy images^21–25^. These methods enable the analysis of whole blood samples using basic instrumentation (e.g., an optical microscope equipped with a camera), reducing the complexity and costs associated with cell quantification. While this approach is promising, it is limited by the small sample volume that can be analyzed from static microscopy images, hindering its use for high throughput analysis. To overcome this issue, Hirotsu et al. developed an AI-based flow cytometer for label-free detection of nucleated cells in peripheral blood samples^26^. In this approach, the sample and two sheath flows were pumped through a microfluidic channel and cells were imaged using quantitative phase microscopy. The resulting images were then classified using a pattern recognition model that was trained with supervised ML. While this platform could differentiate white blood cells from cancer cell lines with similar accuracy as flow cytometry, it required the use of purified blood samples (i.e., RBCs and platelets removed) and a complex experimental setup involving syringe pumps and advanced optical components (HeNe laser, acousto-optic modulators, lenses).

To overcome the limitations described above, we have developed an AI-enabled microfluidic cytometer for rapid quantification of CD4^+^ T cells in whole blood. In this approach, CD4^+^ T cells are labeled with anti-CD4 antibody-coated microbeads using a rapid (5 min) and simple (one-step, wash-free) process, and a video recording of the sample flowing in the microfluidic chip is analyzed using a convolutional neural network (CNN)-based object detection algorithm that has been trained to detect bead-labeled cells in whole blood. The sample is transported through the chip via gravity-driven slug flow, eliminating the need for pumps and simplifying user operation. We show the capability of this platform to quantify CD4^+^ T cells in fingerprick blood samples obtained from healthy donors within 15 min with similar accuracy as flow cytometry (<10% deviation between cell counts determined by both methods). Due to its speed, portability and ease of use, this platform is a promising tool for cell quantification which has potential to be used for point-of-care applications.

## Results

### Design and operation of the microfluidic device

**Fig. 1:**
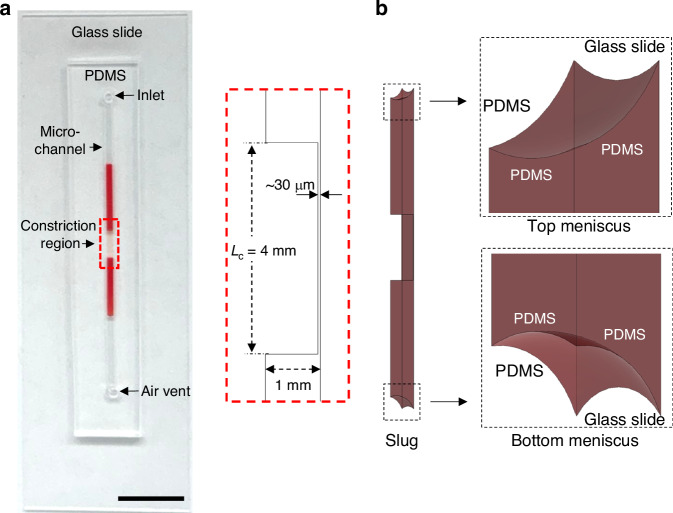
Overview of the microfluidic chip. **a** Photograph of the microfluidic chip containing a 20 µL slug of 10×-diluted blood. Scale bar, 10 mm. Inset shows the cross-section of the constriction region. *L*_*c*_: length of the constriction region. **b** Schematic illustration depicting the liquid slug in the microchannel. Insets show enlarged views of the menisci at the top and bottom of the slug

The microfluidic chip consists of a polydimethylsiloxane (PDMS) microchannel (length = 25 mm, width = 1 mm, height = 1 mm) bonded to a glass microscope slide (Fig. 1a). One end of the microchannel contains the sample inlet and the other end contains an air vent. There is a constriction region (length = 4 mm, width = 1 mm, height = 30 µm) in the middle of the channel, which is where the flow is imaged and recorded. The reduced height of the constriction region enhances cell visualization by reducing the flow rate and confining the cells within the same focal plane to minimize blurring during microscopic imaging. The procedure for bonding the PDMS microchannel to the glass slide involved an oxygen plasma surface treatment, which caused the PDMS surfaces to become hydrophilic (static contact angle, θ = 16.2° ± 2.3°, Fig. S1a). However, the formation of a liquid slug in a microchannel requires the channels surfaces to be hydrophobic^27^. Therefore, the microfluidic chip was heated immediately following PDMS-glass bonding to accelerate the hydrophobic recovery of PDMS^28,29^, resulting in hydrophobic PDMS microchannel surfaces (θ = 81.1° ± 1.8°, Fig. S1b). To evaluate the long-term stability of the plasma- and heat-treated PDMS surface, static contact angle measurements were performed on plasma- and heat-treated PDMS samples that were stored at ambient conditions (temperature = 20 °C, relative humidity = 55%) for varying durations. There was a negligible (<1%) change in the contact angle after 30 days of storage (Fig. S1c, d), suggesting that the microfluidic device can be stored for a moderate amount of time with a negligible impact on its functionality.

Upon dispensing the blood sample into the inlet of the microfluidic chip, it forms a liquid slug due to the surface tension between the sample and the channel walls (Fig. 1b). The surface tension force is a function of the contact angle hysteresis between the advancing contact angle at the bottom meniscus and the receding contact angle at the top meniscus^30^. Based on measurements of the advancing and receding contact angles at the bottom and top menisci of a slug of 10×-diluted blood in the microchannel (Fig. S2), the contact angle hysteresis was determined to be 19.9° ± 3.6°. The gravitational force acting on the slug causes it to be pulled downward through the microchannel where its motion is influenced by the surface tension force resisting the deformation of the air-liquid interfaces and the viscous forces opposing the flow due to shear stresses at the interface between the liquid and channel walls. The balance between the gravitational force, surface tension force and shear force results in the slug moving through the channel at a constant velocity (Video S1). The capillary number (${Ca}=\mu U/\gamma ,$ where *μ* is the dynamic viscosity of liquid, *U* is the average velocity of the slug and *γ* is the surface tension coefficient of the liquid) of this system is 1.8 × 10^−6^, which indicates that surface tension force is dominant over the shear force^31^, causing the slug to maintain concave menisci as it moves through the channel^32^. The concave shape of the menisci at the bottom and top of the slug during slug flow is consistent with observations in prior studies on gravity-driven slug flow in small channels^33,34^. The Bond number (${Bo}={\rho g{D}_{h}}^{2}/\gamma$, where *ρ* is the density of the liquid, *g* is gravitational acceleration, *D*_*h*_ is the hydraulic diameter of the channel and *γ* is the surface tension coefficient of the liquid) in the microfluidic device ranges from 6 × 10^−4^ (constriction region) to 0.17 (main channel), indicating that the gravitational force is comparable to the surface tension force^35^, causing the slug to be driven through the microchannel via gravity. The Reynolds number ($\mathrm{Re}=\rho U{D}_{h}/\mu$, where *ρ* is the density of the liquid, *U* is the average velocity of the slug, *D*_*h*_ is the hydraulic diameter of the channel and *μ* is the dynamic viscosity of liquid) in the device ranges from 0.13 (constriction region) to 0.07 (main channel), indicating that the flow is fully laminar.

### Characterization of gravity-driven slug flow in the microchannel

**Fig. 2:**
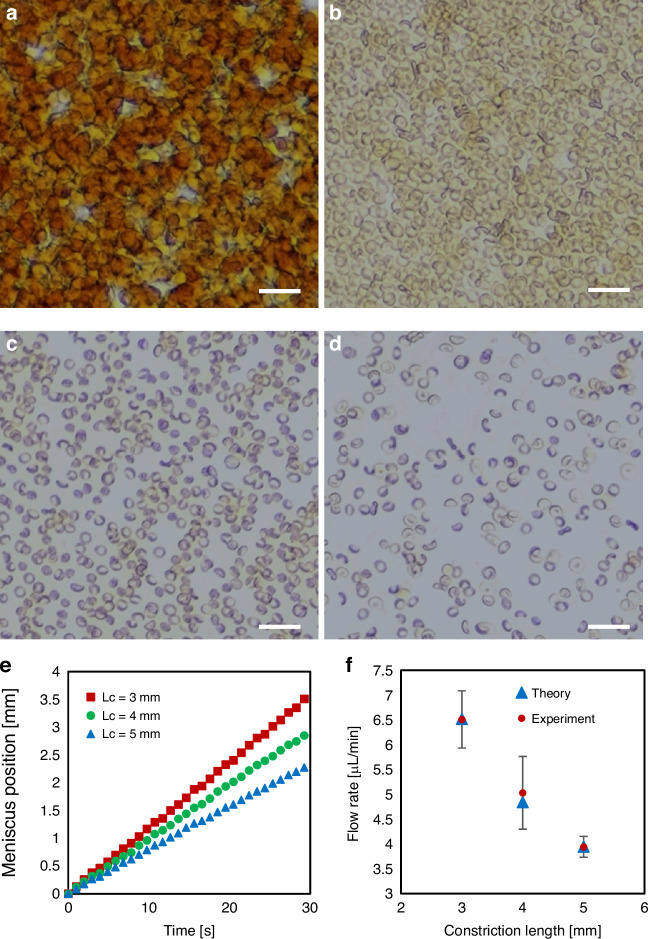
Characterization of gravity-driven slug flow in the microchannel. Optical micrographs of (**a**) undiluted whole blood, (**b**) 5×-diluted blood, (**c**) 10×-diluted blood and (**d**) 20×-diluted blood. Scale bars, 25 µm. **e** Position of the bottom meniscus of the slug vs. elapsed time for microchannels with different constriction lengths. **f** Theoretical and experimental flow rates for microchannels with different constriction lengths. Each data point in the experimental group represents the mean ± standard deviation (SD) of three independent measurements

Whole blood is a densely packed suspension with RBCs and platelets accounting for ~95% of the cellular components^36^, which hinders the ability of ML-based object detection models to accurately identify bead-labeled cells due to crowding and overlapping of cells (Fig. 2a). Therefore, we first evaluated different blood dilutions to determine the optimal dilution factor that would minimize cell overlap for imaging and AI-based cell identification. A 5× dilution resulted in significantly reduced cell overlap, however, the cellular components were still overcrowded (Fig. 2b), which could lead to false-positive or false-negative cell identification. A 10× dilution resulted in a uniform cell distribution with a negligible amount of cell overlap or crowding (Fig. 2c). While further diluting the sample to 20× resulted in a sparser cell distribution with improved cell visibility (Fig. 2d), excessive dilution would require a larger sample volume and longer processing times to obtain accurate cell counts. Thus, 10× was selected as the optimal sample dilution factor and used in all subsequent studies.

We studied the characteristics of gravity-driven slug flow in our device by measuring the time for a slug of 10×-diluted blood to move through microchannels with varying constriction region lengths. For these experiments, video of the slug movement through the constriction region was recorded and the position of the bottom meniscus was tracked over time. For all of the constriction lengths that were studied, the meniscus position exhibited a linear relationship with the elapsed time, indicating that the slug moves at a constant velocity in the microchannel (the slope of the line represents the velocity of the slug) (Fig. 2e). For each of these channel geometries, the theoretical volumetric flow rate was calculated (Fig. S3 and equations 1–5 in Supplementary Information) and compared with the flow rates measured experimentally. The theoretical flow rates matched closely with the experimental flow rates (values were within 3.7%), validating the accuracy of the theoretical analysis (Fig. 2f). Furthermore, we observed that the flow rate was inversely correlated with the length of the constriction region where a longer constriction length resulted in a slower flow velocity, as expected. The gravitational force and the surface tension force remain constant with different constriction lengths since the sample volume and surface properties do not change. Thus, for microchannels with a longer constriction region, the slug experiences a higher viscous shear force compared to a microchannel with a shorter constriction region, resulting in a slower flow rate. Considering the ideal flow rate needed for accurate image-based cell identification with minimal cell sedimentation, 4 mm was selected as the optimal length of the constriction region. All subsequent experiments and particle/cell quantification measurements were performed using microfluidic devices with fixed channel dimensions (Fig. 1a). From our theoretical analysis, the flow rate is dependent on the channel dimensions, fluidic properties of the sample and gravitational acceleration, which are all fixed in our system (equation 5 in Supplementary Information). Therefore, changing the sample volume will affect the gravitational force acting on the liquid slug, resulting in a different flow rate. For example, dispensing a larger amount of sample in the device would generate a longer slug, which would experience a larger gravitational force and cause it to flow at a faster velocity. A sample volume of 20 µL (10×-diluted blood) was selected for particle/cell quantification measurements, which flowed through the microchannel within 3 min, enabling rapid sample analysis.

### Optimization of CD4^+^ T cell-bead binding

**Fig. 3:**
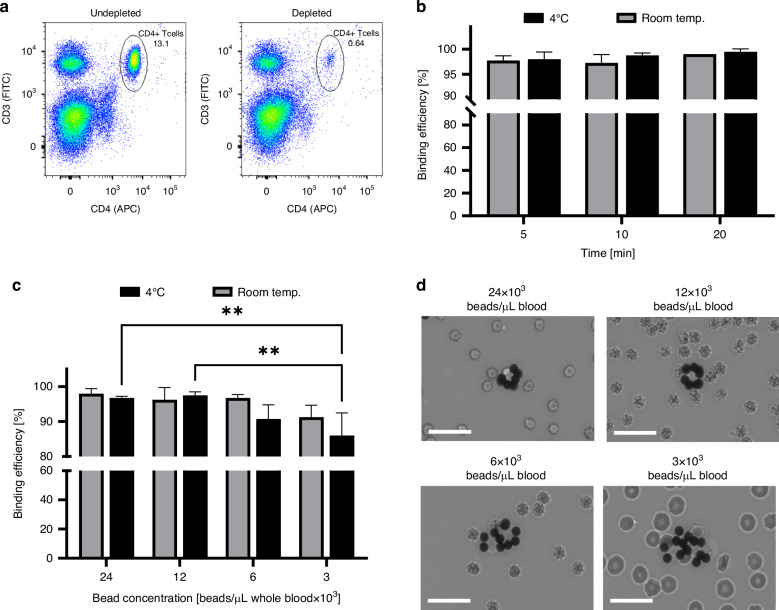
Optimization of CD4^+^ T cell-bead binding. **a** Representative dot-plots of undepleted blood samples and CD4^+^ cell-depleted blood samples using anti-CD4 antibody-coated beads analyzed via flow cytometry. **b** Binding efficiency of anti-CD4 antibody-coated beads to CD4^+^ cells at varying incubation times and temperatures at a bead concentration of 24 × 10^3^ bead/μL of whole blood. Each bar represents the mean ± SD, *n* = 4. **c** Binding efficiency of anti-CD4 antibody-coated beads to CD4^+^ cells using varying bead concentrations and incubation temperatures for 5 min. Each bar represents the mean ± SD, *n* = 3–4. **P < 0.01 (two-way ANOVA with Tukey’s multiple comparisons). **d** Optical micrographs of 10×*-*diluted blood samples after room temperature incubation with anti-CD4 antibody-coated beads at varying bead concentrations for 5 min. Scale bars, 25 μm

Experiments were performed to optimize the CD4^+^ T cell-bead labeling efficiency. For these studies, whole blood samples were incubated with or without anti-CD4 antibody-coated beads and the resulting CD4^+^ cell-depleted samples were analyzed using flow cytometry (Fig. 3a). We first investigated the influence of incubation time and temperature on the cell-bead binding efficiency. CD4^+^ cells were depleted from whole blood at rates of >97% with no statistical differences for incubation times of 5, 10 or 20 min at either room temperature or 4 °C (Fig. 3b). Thus, 5 min was selected as the optimal incubation duration to shorten the overall assay time. Next, the effect of bead concentration on the cell binding efficiency was assessed at room temperature and 4 °C. Again, CD4^+^ cells were depleted from the blood samples at very high rates ranging from 86 to 98%. A two-way ANOVA demonstrated that both the incubation temperature (*P* < 0.05) and the bead concentration (*P* < 0.0001) had a significant effect on binding efficiency. The effect of bead concentration can be seen by comparing samples incubated at 4 °C as the two highest concentrations (24 × 10^3^ and 12 × 10^3^ beads/μL of whole blood) resulted in significantly higher (>10%) binding efficiency compared to the lowest bead concentration (3 × 10^3^ beads/μL of whole blood) (Fig. 3c). For bead incubation performed at room temperature, there was not a significant improvement between samples incubated at different bead concentrations. Thus, we decided to perform bead incubation at room temperature to eliminate the need for refrigeration to simplify the assay workflow.

Blood samples were imaged via microscopy following bead incubation at room temperature to visualize bead-cell binding. Interestingly, cell and bead aggregation was observed for samples with bead concentrations of 6 × 10^3^ and 3 × 10^3^ beads/μL of whole blood (Fig. 3d). The aggregates are likely formed by the CD4^+^ cells acting as nexus points between anti-CD4 antibody-coated beads. In support of this hypothesis, samples with higher bead concentrations of 24 × 10^3^ and 12 × 10^3^ bead/μL of whole blood (which have higher cell-to-bead ratios) exhibit reduced aggregation, resulting in single CD4^+^ cells being bound to several beads. This morphology of a single cell bound to 3 or more beads is preferable to large aggregates of beads and cells as it enhances the identification of individual bead-labeled cells by the ML model. For these reasons, 12 × 10^3^ beads/μL of whole blood was selected as the optimal bead concentration.

In addition to CD4^+^ T cells, other cells, such as monocytes and macrophages, are known to express CD4 but lack CD3 which is expressed by T cells. As this platform uses a single marker (anti-CD4 antibody) to identify CD4^+^ T cells, we sought to determine if non-CD4^+^ T cells were binding the antibody-coated beads in whole blood. It was found that 83.0% ± 0.2% and 83.1 ± 0.4% of the bound cells were CD4^+^ T cells, while the remaining cells were CD4^+^ but CD3^−^ in the 24 × 10^3^ and 12 × 10^3^ bead/μL of whole blood sample groups, respectively (Fig. S4). This result shows that the anti-CD4 antibody-labeled beads did not exclusively label CD4^+^ T cells, indicating that CD4^+^CD3^−^ cells could potentially affect the CD4^+^ T cell counts generated by the AI-enabled microfluidic cytometer. However, as described later in *Quantification of CD4*^*+*^ *T cells in human blood samples*, CD4^+^CD3^−^ cell levels did not affect the accuracy of this platform.

### Device characterization using human blood spiked with polystyrene particles

**Fig. 4:**
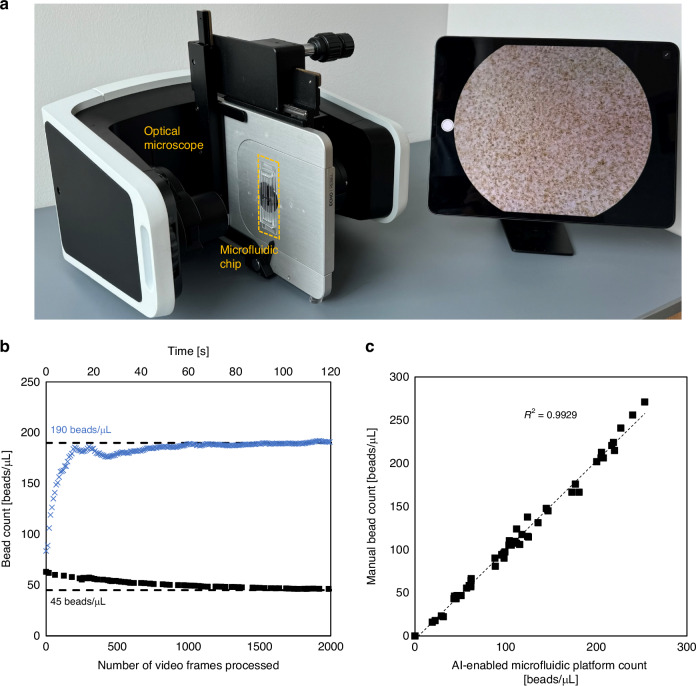
Characterization of the AI-enabled microfluidic cytometer using human blood samples spiked with polystyrene particles. **a** Photograph of the experimental setup for particle and cell quantification measurements. **b** Counts determined by the AI-enabled microfluidic cytometer when analyzing 10×-diluted blood samples spiked with 10 µm polystyrene particles at a concentration of 45 beads/µL and 190 bead/µL vs. number of video frames processed by the ML model and the corresponding processing time. **c** Counts of 10×-diluted blood samples spiked with varying concentrations of 10 µm polystyrene particles determined using the AI-enabled microfluidic cytometer and manual counting. Each data point represents a single measurement from a unique sample, *n* = 55

We first characterized the AI-enabled microfluidic cytometer by evaluating its performance in quantifying 10 µm polystyrene particles, which were used to simulate CD4^+^ T cells, spiked in 10×-diluted whole blood. The experimental setup for performing particle and cell quantification measurements consisted of the microfluidic chip and an optical microscope equipped with a camera (Fig. 4a). The microfluidic chip was secured to the stage and positioned so that the objective was focused on the constriction region of the microchannel. For these device characterization studies, the ML model was trained using annotated images obtained from separate video recordings of blood samples spiked with varying concentrations of polystyrene particles flowing through the microfluidic chip, ensuring that the training dataset was distinct from the samples used for quantification measurements. To determine the minimal sample volume needed to yield accurate and reliable cell counts, blood samples spiked with 10 µm polystyrene particles at a low (45 particles/µL) and high (190 particles/µL) concentration were analyzed using the AI-enabled microfluidic cytometer. As shown in Fig. 4b, the particle count fluctuates when less than ~700 frames are processed due to the count being averaged over a smaller number of video frames. As more video frames are processed, the fluctuations in the count diminishes and the accuracy of the ML model increases. The particle count determined by the AI-enabled microfluidic cytometer converges to the actual (spiked) particle concentration after processing ~2,000 frames, which corresponds to a processing time of ~133 s (at 15 frames per s [fps]). Based on the flow rate (5 µL/min) at which the slug moves through the microchannel, this processing time corresponds to a volume of 11.1 μL, which is the minimal sample volume needed to obtain an accurate quantification measurement. To account for any losses in the sample due to sample handling (e.g., pipetting), 20 µL was selected as the optimal volume of 10×-diluted blood for cell quantification measurements, corresponding to a (undiluted) whole blood volume of 2 µL, which can be readily obtained from a fingerprick.

The detection range of the AI-enabled microfluidic cytometer was evaluated by analyzing 55 blood samples spiked with varying concentrations of polystyrene particles ranging from 19 to 250 particles/µL. Particle counts determined by the AI-enabled microfluidic cytometer were compared to those obtained from manual counts. The particle count determined by both methods are plotted in a scatter plot (Fig. 4c) and linear regression analysis showed that particle counts determined by this platform were highly correlated (Pearson’s correlation coefficient, *R*^2^ = 0.9929) with those determined by manual counting over a large range of concentrations from 19 to 250 particle/µL, demonstrating the capability of the AI-enabled microfluidic cytometer to accurately quantify cell-sized particles in whole blood.

### Quantification of CD4^+^ T cells in human blood samples

**Fig. 5:**
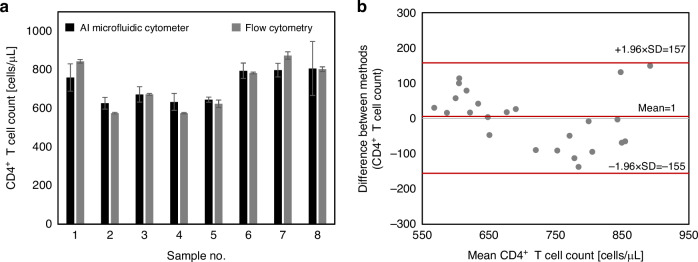
Performance of AI-enabled microfluidic cytometer for CD4^+^ T cell quantification in human blood. **a** CD4^+^ T cell counts obtained from 8 blood samples measured using the AI-enabled microfluidic cytometer and flow cytometry. Each bar represents the mean ± SD of three technical replicates, *n* = 3. **b** Bland-Altman plot (AI-enabled microfluidic cytometer count—flow cytometry count vs. mean count) showing the agreement between the AI-enabled microfluidic cytometer and flow cytometry for quantifying CD4^+^ T cells. Each data point represents a single measurement where measurements for each of the 8 blood samples were performed in triplicates

To evaluate the analytical performance of the AI-enabled microfluidic cytometer for quantifying CD4^+^ T cells, fingerprick blood samples from 8 unique donors were analyzed using the AI-enabled microfluidic cytometer and flow cytometry. These blood samples were freshly collected for this study and distinct from the samples that were used to generate the training and validation datasets for the ML model. Video S2 shows a magnified view of a blood sample flowing through the constriction region of the microfluidic chip, which reveals the capability of the ML algorithm to rapidly identify bead-labeled CD4^+^ T cells. CD4^+^ T cell counts generated by this platform were closely correlated to cell counts obtained from flow cytometry (<10% deviation between both methods), validating the accuracy of the AI-enabled microfluidic cytometer (Fig. 5a). A Bland-Altman analysis resulted in a slight positive count bias of 1 cells/μL for the AI-enabled microfluidic cytometer (compared to flow cytometry) and narrow limits of agreement between −155 and 157 cells (d – 1.96 SD to d + 1.96 SD).

Lastly, we investigated the effect of the presence of CD4^+^CD3^−^ cells in blood on the accuracy of CD4^+^ T cell counts measured by the AI-enabled microfluidic cytometer. Using flow cytometry, the concentration of CD4^+^CD3^−^ cells in each of the blood samples was determined to be between 190 and 315 cells/μL. The difference between the CD4^+^ T cell levels measured by the AI-enabled microfluidic cytometer and flow cytometry was calculated and plotted against the CD4^+^CD3^−^ cell levels (Fig. S5). If non-specific binding of anti-CD4 antibody-coated beads was occurring, a correlation between the number of CD4^+^CD3^−^ cells and difference between quantification methods would be expected. However, no correlation (coefficient of determination, *R*^*2*^ = 0.004) was observed, indicating that CD4^+^CD3^−^ cell levels have a negligible effect on the accuracy of the AI-enabled microfluidic cytometer for the range of CD4^+^CD3^−^ cell levels tested.

## Discussion

Conventional flow cytometry-based techniques for quantifying cell subpopulations in blood are time-consuming (>1 h), involve complicated sample preparation and processing protocols, and require the use of bulky and expensive instrumentation. Impedance-based flow cytometers have been developed that are faster (5–20 min) and involve simpler sample preparation procedures, however, are limited by the need for an expensive impedance analzyer^10–13^. The AI-enabled microfluidic cytometer presented in this work offers a rapid 15 min turnaround time while requiring minimal sample preparation and the use of an optical microscope equipped with a camera, which is relatively inexpensive and widely accessible. A comparison of the AI-enabled microfluidic flow cytometer with impedance flow cytometry and conventional flow cytometer is presented in Table S1. Several unique strategies were implemented in the development of this platform. First, the blood sample is driven though the microfluidic chip via gravity-based slug flow, eliminating the need for a pump and simplifying user operation. The hydrodynamics of slug flow in microchannels has previously been studied and the vast majority of these reports have focused on pressure-driven flow involving a train of liquid slugs in air^37^. Studies analyzing the physics of gravity-driven slug flow involving a single slug of water in a circular capillary have also been reported^31,34^. In this work, we employ gravity-driven slug flow to drive a blood sample through a microchannel at a constant velocity, which is crucial for achieving accurate cell detection using an AI-based object detection technique. To the best of our knowledge, this is the first time that gravity-driven slug flow has been employed for processing or analyzing biofluid samples.

In our approach, the target CD4^+^ T cells are labeled with anti-CD4 antibody-coated beads and bead-labeled cells are identified using a trained CNN-based object detection algorithm (YOLO(v5)), enabling highly specific, high-throughput cell quantification in whole blood. While other CNN-based object detection algorithms are available, YOLO(v5) was selected for this application due to its high accuracy and high inference speed (129–248 fps)^38^, allowing for rapid and high-throughput image processing. This offers advantages over prior AI- and non-AI-based flow cytometry techniques that involve lengthy and complicated sample preparation and processing protocols, such as blood separation and RBC lysis, making this platform faster and simpler to operate. While this technique requires the target cells to be labeled with antibody-coated beads, the labeling procedure used in this approach is simple (one-step, wash-free), rapid (5 min) and can be performed with whole blood.

Experiments to evaluate the performance of the AI-enabled microfluidic cytometer revealed its ability to quantify CD4^+^ T cells in human blood samples with similar accuracy as flow cytometry (<10% deviation between the two methods), while being at least 4× faster and simpler to operate. Furthermore, the detection range of this platform (19 to 250 particles/µL in 10×-diluted blood) corresponds to 190 to 2500 cells/µL in undiluted whole blood, which encompasses clinically relevant levels of CD4^+^ T cells in healthy individuals (430–1740 cells/µL)^39,40^, persons infected with HIV who are undergoing ART ( ≤ 350 cells/µL)^41^ and individuals with an increased risk of non-AIDS defining cancers (350–499 cells/µL), including Kaposi’s sarcoma and non-Hodgkin lymphoma^6^. Currently, this platform has only been tested using blood samples obtained from healthy donors and further studies will be needed involving the analysis of blood samples from other populations of individuals, such as HIV patients, to further assess the diagnostic utility of this platform.

In this work, proof of principle was demonstrated by analyzing human blood samples for quantifying CD4^+^ T cells, which has clinical relevance as a diagnostic/prognostic tool for assessing immune status. This AI-enabled microfluidic cytometer can be readily modified to quantify other cell subpopulations by replacing the anti-CD4 antibody-coated beads with beads that are coated with antibodies targeting other proteins expressed on different cell types. Furthermore, we envision that this platform can be modified for multiplexed cell quantification by using different colored antibody-coated beads. For example, CD4^+^ T cells and CD19^+^ B cells could be simultaneously quantified by using red-colored anti-CD4 antibody-coated beads and blue-colored anti-CD19 antibody-coated beads and retraining the ML algorithm with images containing both types of bead-labeled cells. Lastly, the use of a compact (e.g., smartphone-based) microscope would enhance the portability of this technique, further increasing the accessibility of this device for diagnostic testing in remote and resource-limited settings.

## Materials and Methods

### Chemicals

Dipotassium ethylenediaminetetraacetic acid (EDTA) was purchased from Sigma-Aldrich (St. Louis, MO). 10× phosphate buffered saline (PBS) and bovine serum albumin (BSA) were purchased from Fisher Scientific (Hampton, NH). Brilliant violet 570 anti-human CD45 (304034) and APC anti-human CD4 (300552) were purchased from BioLegend (San Diego, CA), and FITC anti-human CD3 (555916) was purchased from BD Biosciences (Franklin Lakes, NJ).

### Fabrication of the microfluidic chip

The PDMS microchannel was fabricated using soft lithography. Briefly, the master mold was designed using SOLIDWORKS software (SolidWorks Corp, Waltham, MA) and fabricated in polymethyl methacrylate (PMMA) (McMaster-Carr, Elmhurst, IL) using a CNC Mini-Mill/3 (Minitech Machinery Corp., Norcross, GA). PDMS pre-polymer and curing agent (SYLGARD™ 184 Silicone Elastomer, The Dow Chemical Company, Midland, MI) were mixed at a 10:1 ratio, poured onto the PMMA master mold and cured at 80°C for 3 h. After curing, the PDMS microchannel was demolded and holes for the inlet and air vent were punched using a 15-gauge needle tip. The PDMS microchannel was then plasma treated (300 W for 20 s) and bonded to a glass microscope slide (Fisherbrand, Fisher Scientific). Immediately after PDMS-glass bonding, the microfluidic chip was placed on a hot plate at 90 °C for 48 h for heat treatment.

### Blood sample collection

Blood samples were collected from healthy donors with informed consent under a Rice University IRB-approved protocol (IRB-FY2021-147). Blood was obtained via fingerstick using a lancing device (Bayer Microlet) and 30 G lancets (CareTouch), and collected in a 1.5 mL microcentrifuge tube containing 1 μL of EDTA (150 mg/mL). Blood samples were stored on ice for no more than 2 h until analysis.

### CD4^+^ T cell-bead binding optimization

CD4 antibody-coated microbeads (Dynabeads, Thermo Fisher Scientific, Waltham, MA) were added at various concentrations to a 1.5 mL microcentrifuge tube and washed with 1 mL of isolation buffer composed of 1 L of 1× PBS with 1 g of BSA. The tube was placed on a magnetic rack and the isolation buffer was removed. Beads were immediately resuspended in 50 μL of whole blood. Tubes were then allowed to incubate at room temperature ( ~ 23 °C) or 4 °C for varying durations on a rotisserie shaker (Barnstead Thermolyne Corporation, Ramsey, MN). After incubation, unbound cells were removed by washing the beads 3 times with 1 mL of isolation buffer. Beads were separated using a magnetic rack. The removed solution was then centrifuged at 500 rcf for 5 min and the supernatant was removed. The remaining cells were resuspended in 50 μL of PBS and immediately prepared for flow cytometry. Blood (without bead labeling) was used as an undepleted sample for comparison with the depleted sample to evaluate the cell binding efficiency. CD4^+^ T cells were identified as staining positive for both CD4 and CD3 surface markers, while non-specific monocyte binding was quantified as positive for CD4 expression and negative for CD3 expression.

### Training and deployment of the ML algorithm

The YOLO(v5) object detection model was trained and validated using a dataset of ~4,500 annotated images (Fig. S6a). Images for the dataset were generated from video recordings of 10×-diluted blood sample containing polystyrene particles or bead-labeled cells flowing through the microchannel at different operating conditions (e.g., varying bead concentrations, varying levels of brightness, etc.) (Fig. S7). Each video frame (1920 × 1440 pixels) was segmented into 9 images (640 × 640 pixels) with overlapping edges. Images were visually examined to identify particles or bead-labeled cells and manually annotated using Label Studio software (Fig. S8). Of the ~4,500 annotated images, 80% were used for training and the remaining 20% was used to validate the model. The training process was carried out over 250 epochs on a T4 GPU (Google Collaboratory). A data augmentation technique was used to alter key features in the images, including the particle orientation, size, and lighting conditions, to improve the model’s robustness. The augmentation parameters included hue, saturation variation, rotation, translation, scaling, shear, vertical and horizontal flip and mosaic. The training culminated with an F1 score (geometric mean of recall and precision value) of 0.90 with at a confidence score of 0.35. After training and validation, the model was deployed to process video frames from recordings of freshly collected blood samples obtained from healthy donors using the T4 GPU (Google Collaboratory). The inference data from each frame (e.g., the centroid coordinates and the corresponding box bounding each polystyrene particle or bead-labeled CD4^+^ T cell) was stored as a. txt file for downstream analysis, which included aggregating the particle/cell detection events across all frames, applying the predefined probability threshold and size-based filtering followed by quantification. A probability threshold of 60% was used for the inference of particles and 35% was used for the inference of bead-labeled CD4^+^ T cells in video frame images, which were determined based on the maximum F1 scores obtained during the training process. Particle/cell detection events outside of 2 SD (based on the size of the bounding box) were removed to minimize false positive results. The particle/cell count was calculated based on the ratio of the mean count per frame that contained at least one particle/cell to the sample volume in each frame. For quantification measurements below 40 particles/µL, the particle count was calculated based on the ratio of the mean count per frame to the sample volume per frame to account for samples with fewer than one particle per frame. The inference speed varied between 35 and 55 ms per frame (based on a frame size of 1920 × 1440 pixels), where 2,500 frames were processed in ~140 s. Subsequent reading of individual detection files and quantification of the results was completed in ~180 s.

### Particle and CD4^+^ T cell quantification measurements

Particle and cell counts were performed using a Rebel optical microscope (Discover Echo Inc., San Diego, CA). The microfluidic chip was secured to the microscope stage and positioned so that the objective was focused on the constriction region. The microscope was placed in a horizontal configuration, resulting in the microfluidic chip to be positioned in a vertical orientation. For cell quantification measurements, prewashed anti-CD4 antibody-labeled microbeads were added to 50 µL of whole blood to a final concentration of 12 × 10^3^ beads per μL of blood, and incubated for 5 min at room temperature. The bead-blood sample was diluted to 10× using isolation buffer and 20 µL of the diluted bead-blood sample was then dispensed into the inlet of the microfluidic chip. For particle quantification measurements, polystyrene particles were added to 50 µL of 10×-diluted whole blood, and 20 µL of the particle-blood sample was dispensed into the microfluidic chip. Video of the sample flowing through the microchannel was recorded using the microscope camera. The captured videos were transferred to a computer and processed using the trained model via a T4 GPU (Google Collaboratory) (Fig. S6b).

### Flow cytometry

Whole or depleted blood samples (50 µL) and 2 μL of Human TruStainFcX^TM^ (422301, Biolegend) were added to 5 mL centrifuge tubes and incubated on ice on an orbital shaker for 5 min. A total of 50 μL of antibody solution was then added to the tube and incubated on ice (protected from light) for an additional 30 min. For cell depletion studies, the antibody solution consisted of 39 μL of eBioscience^TM^ Flow Cytometry Buffer (Thermo Fisher Scientific), 1 μL of CD4 antibody, and 10 μL of CD3 antibody. A three-color panel consisting of 36.5 μL of staining buffer, 1 μL of CD4 antibody, 10 μL of CD3 antibody, and 2.5 μL of CD45 antibody was used for quantifying CD4^+^ T cell levels for comparison with the AI-enabled microfluidic cytometer, as this panel is more accepted for clinical determination of CD4^+^ T cell counts^42,43^. Next 2 mL of 1× RBC cell lysis buffer (Fisher Scientific) was added to each sample and incubated (protected from light), at room temperature for 10 min, followed by centrifugation at 500 rcf for 5 min. The supernatant was removed and cells were washed 2 times with 1 mL of staining buffer. Cells were resuspended in 950 μL of staining buffer and 50 μL of CountBright^TM^ Pulse Absolute Counting Beads (ThermoFisher). Finally, cells were passed through a 35 μm cell strainer (Corning Inc., Corning, NY) and run on a Sony MA900 flow cytometer (Sony Biotechnology, San Jose, CA). All gates were determined based on fluorescence minus one controls. At least 5000 counting beads were quantified for each sample. CD4^+^ T cell counts were calculated based on the processed sample volume calculated using the known concentration of counting beads.

## Supplementary information

Supplementary Information (clean)

Supplementary Video S1

Supplementary Video S2

## Data Availability

All data are available in the main text or supplementary materials. Additional information within reason is available from the corresponding author.
