# Supplementary material for: Artificial intelligence-enabled microfluidic cytometer using gravity-driven slug flow for rapid CD4+ T cell quantification in whole blood
Source: Microsyst Nanoeng. 2025 Feb 28;11:36. doi: 10.1038/s41378-025-00881-y (PMC11868388; doi:10.1038/s41378-025-00881-y)
Supplement: Supplementary file 1 — Supplementary Information (clean) [file 41378_2025_881_MOESM1_ESM.pdf]

Supplementary Information

Artificial intelligence-enabled microfluidic cytometer using gravity-driven slug flow for rapid CD4$^+$ T cell quantification in whole blood

Desh Deepak Dixit[1†], Tyler P. Graf[2†], Kevin J. McHugh [2,3] and Peter B. Lillehoj[1, 2]*

[1]Department of Mechanical Engineering, Rice University, Houston, TX 77005, USA
[2]Department of Bioengineering, Rice University, Houston, TX 77030, USA
[3]Department of Chemistry, Rice University, Houston, TX 77030, USA

†Indicates that these authors contributed equally to this work
*Correspondence and request for materials should be addressed to: P.B.L.

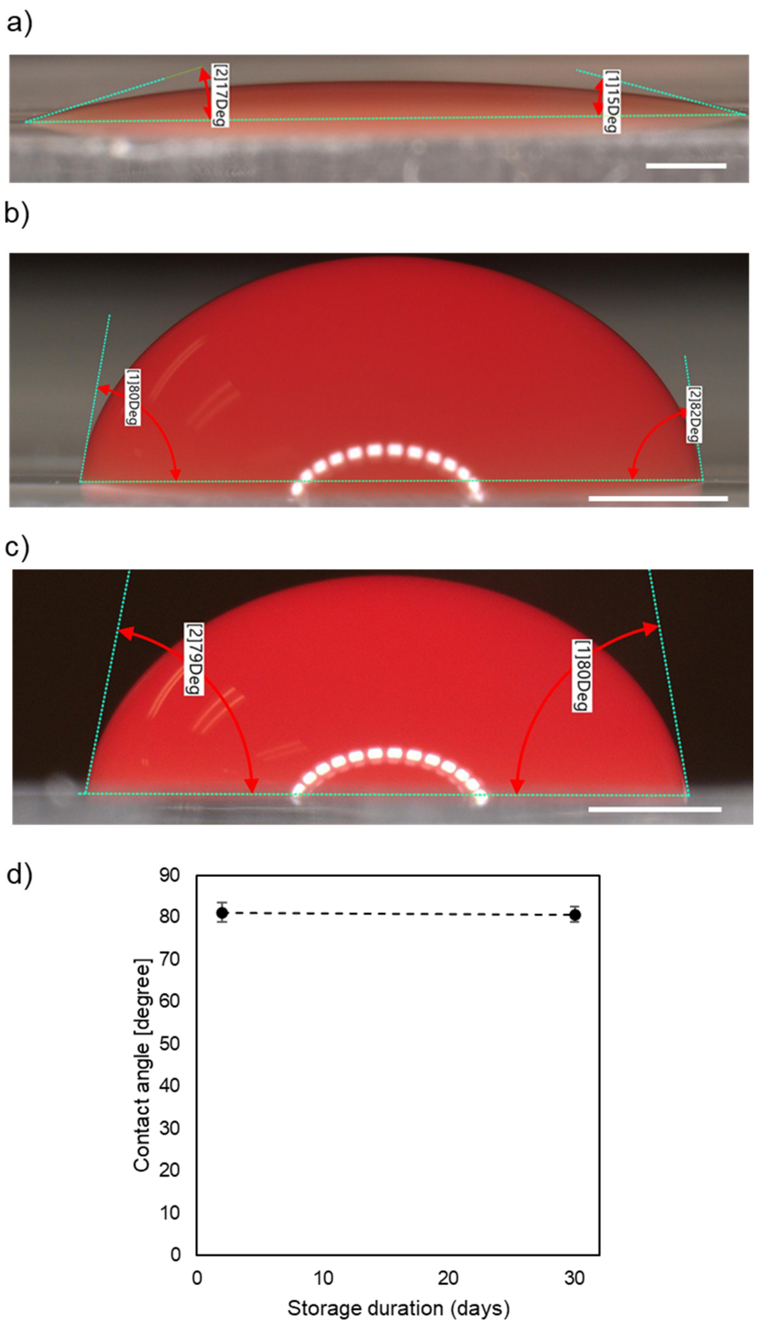

Fig. S1: Static contact angle measurements on plasma-treated PDMS surfaces without and with heat treatment. Optical image of a 20 µL droplet of 10×-diluted blood on (**a**) plasma-treated PDMS, (**b**) plasma-treated PDMS with heat treatment, and (**c**) plasma-treated PDMS with heat treatment after 30 days of storage at ambient conditions. Scale bar, 1000 µm. (**d**) Long-term stability of plasma-treated PDMS with heat treatment. Each data point represents the mean ± standard deviation (SD) of three independent measurements.

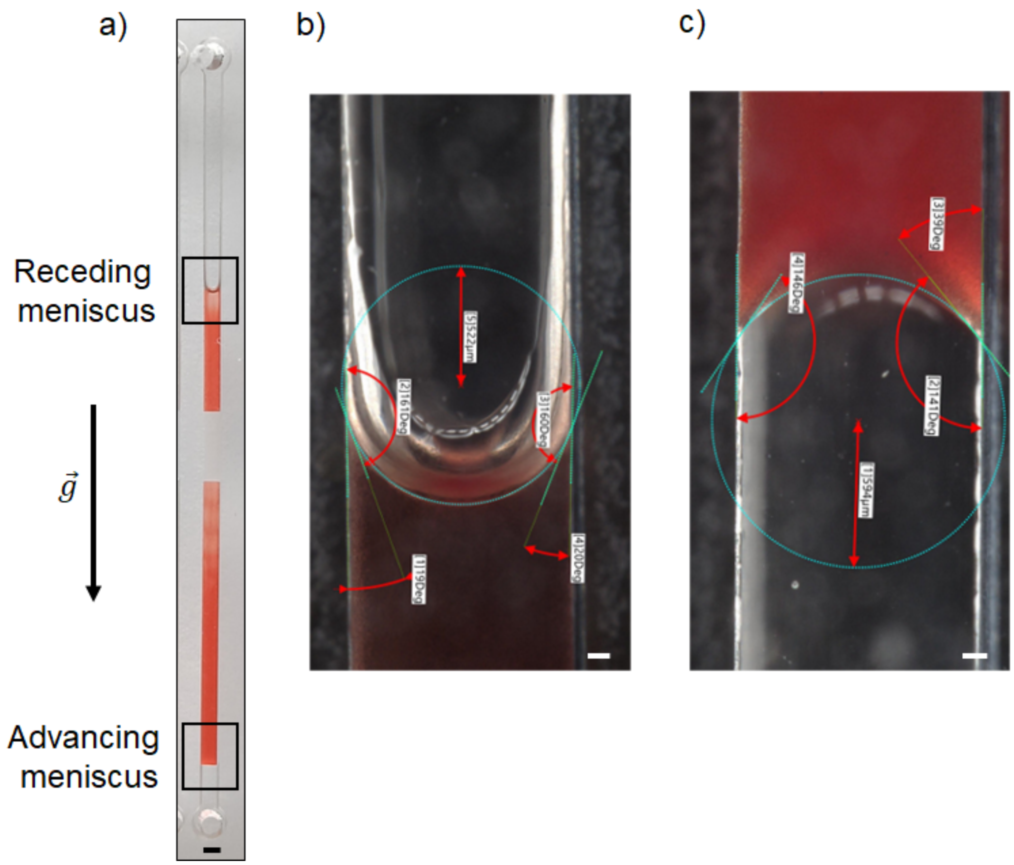

Fig. S2: Advancing and receding contact angle measurements inside the microchannel. (**a**) Optical image of a slug of 10×-diluted blood inside the microchannel. Scale bar, 1 mm. Magnified view of (**b**) the receding meniscus at the top of the slug and (**c**) the advancing meniscus at the bottom of the slug. Scale bars, 100 µm.

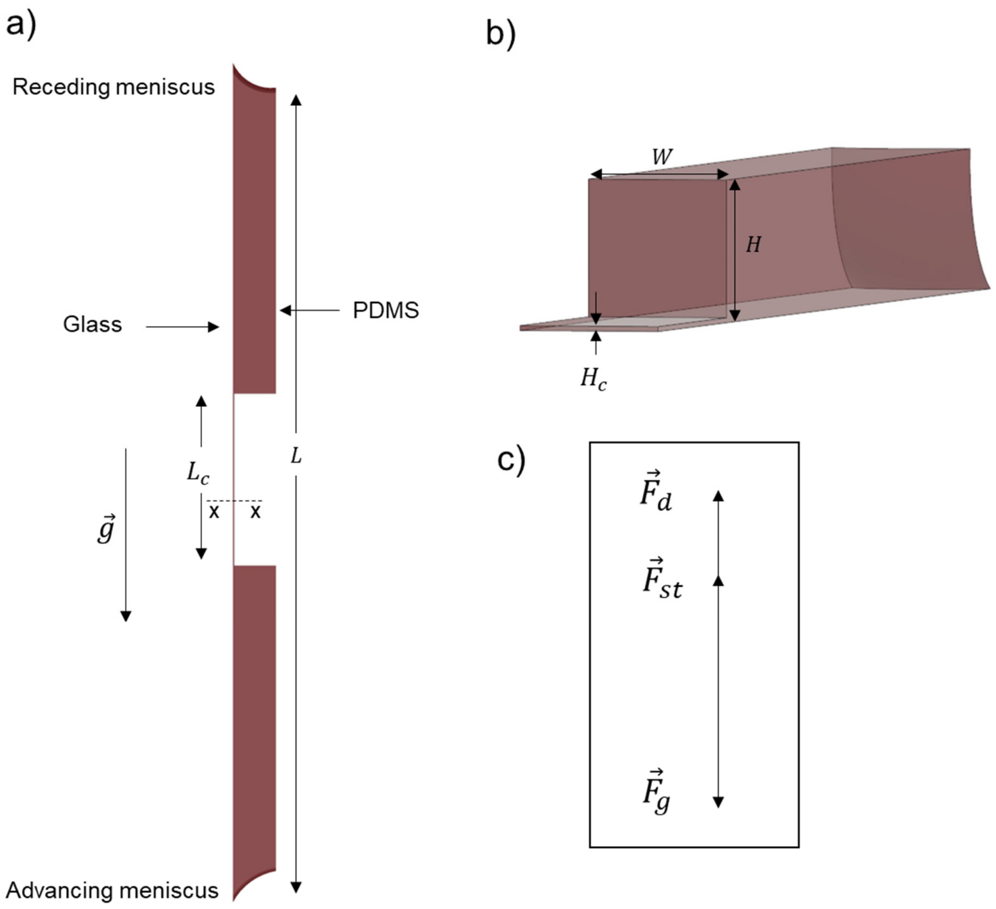

Fig. S3: Theoretical analysis of gravity-driven slug flow inside the microchannel. Schematic illustrations showing (**a**) the side view of the liquid slug and (**b**) the cross-sectional view of the constriction region. (**c**) Free body diagram of forces acting on the liquid slug during gravity-driven flow.

Calculation of theoretical volume flow rate of gravity-driven slug flow in the microchannel

The motion of a liquid slug in a vertical microchannel is governed by the balance of the gravitational force F_g, drag force F_d and surface tension force F_{st} acting on the slug, as represented in **Eq. 1**. F_g is equal to weight of slug (neglecting the menisci), calculated using **Eq. 2**, where ρ is the density of the liquid, g is gravitational acceleration, W, H and L are the width, height and length of the slug, respectively, and W, H_C, and L_C are the width, height and length of the constriction region. F_{st} is calculated by multiplying the cross-sectional area with the Laplace pressure at the receding and advancing menisci using **Eq. 3**[1], where γ is the surface tension

coefficient of the liquid, and $R_{Receding}$ and $R_{Advancing}$ are the radii of curvature at the receding and advancing menisci, respectively. The curvature at the menisci was measured experimentally as shown in **Fig. S2**. F_d is calculated by multiplying the cross-sectional area with the pressure drop (due to the viscous effects) in the channel, as represented in **Eq. 4**[2], where μ is the dynamic viscosity of liquid, ρ is the density of the liquid, Q is the volumetric flow rate, W, H and L are the width, height and length of the slug, respectively, and W, H_C and L_C are the width, height and length of the constriction region. Values for μ and ρ were calculated using the volumetric average. The density, viscosity and surface tension coefficient of the liquid (whole blood diluted 10× with 1× PBS containing 1 g of BSA and 4 mM of Tween 20) were calculated using the following fluid properties: ρ_{PBS} = 1.005 g/mL[3], ρ_{blood} = 1.05 g/mL[4], μ_{PBS} = 1 cP[3], μ_{blood} = 3.5 cP[5], γ_{PBS} = 57 mN/m[6] and γ_{blood} = 55.9 mN/m[7]. By rearranging **Eqs. 1-4**, the volume flow rate can be expressed as a function of the channel dimensions (W, H, L), fluid properties (ρ, μ, γ, R_A, R_R) and gravitational acceleration g, as shown in **Eq. 5**.

$$F_d + F_{st} = F_g \tag{1}$$

$$F_g = \rho g W H (L - L_c) + \rho g W H_c L_c \tag{2}$$

$$F_{st} = WH\gamma \left(\left({}^{2}/_{R_{Receding}} \right) - \gamma \left({}^{2}/_{R_{Advancing}} \right) \right) \tag{3}$$

$$F_d = \left(\frac{12\mu L_c}{{H_c}^3 W} \frac{Q}{\left(1 - 0.63\frac{H_c}{W}\right)} + \frac{12\mu L}{H^3 W} \frac{Q}{\left(1 - 0.63\frac{H}{W}\right)} \right) WH \tag{4}$$

$$Q = \frac{\left((\rho g W H (L - L_c) + \rho g W H_c L_c) - \left(WH\gamma \left(\left({}^{2}/_{R_{Receding}} \right) - \gamma \left({}^{2}/_{R_{Advancing}} \right) \right) \right) \right)}{\left(\frac{12\mu L_c}{{H_c}^3 W} \frac{1}{\left(1 - 0.63\frac{H_c}{W}\right)} + \frac{12\mu L}{H^3 W} \frac{1}{\left(1 - 0.63\frac{H}{W}\right)} \right) WH} \tag{5}$$

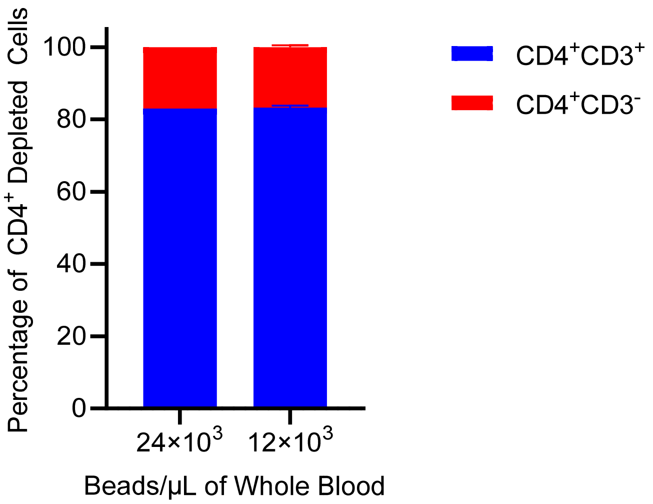

Fig. S4: Flow cytometry analysis of depleted cells expressing $CD4^+$ and $CD3^+/CD3^-$. Percentage of blood cell types depleted using anti-CD4 antibody-coated beads at a bead concentration of $24{\times}10^3$ or $12{\times}10^3$ bead/µL of whole blood after room temperature incubation for 5 min. Each bar represents the mean ± SD, n=3-4.

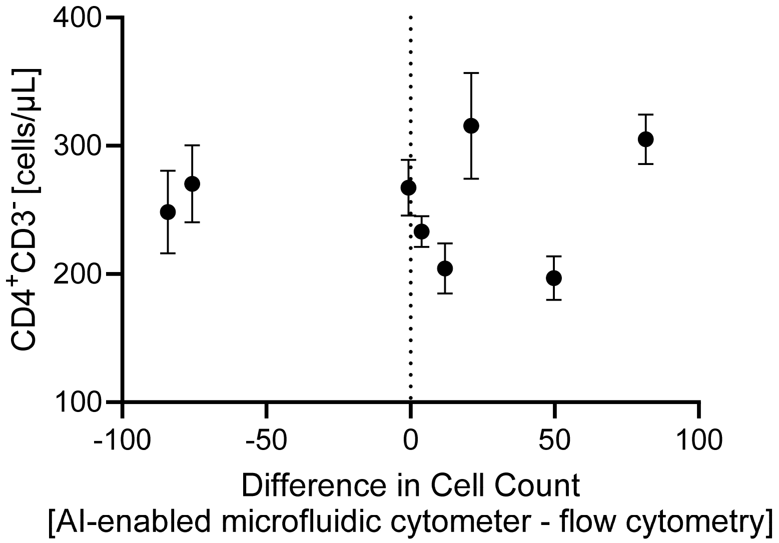

Fig. S5: Influence of $CD4^+CD3^-$ cells on the accuracy of $CD4^+$ T cell count measurements. $CD4^+CD3^-$ cell counts determined by flow cytometry vs. the difference between the $CD4^+$ T cell counts determined by the AI-enabled microfluidic cytometer and flow cytometry. Each data point represents the mean ± SD of three technical replicates, n=3.

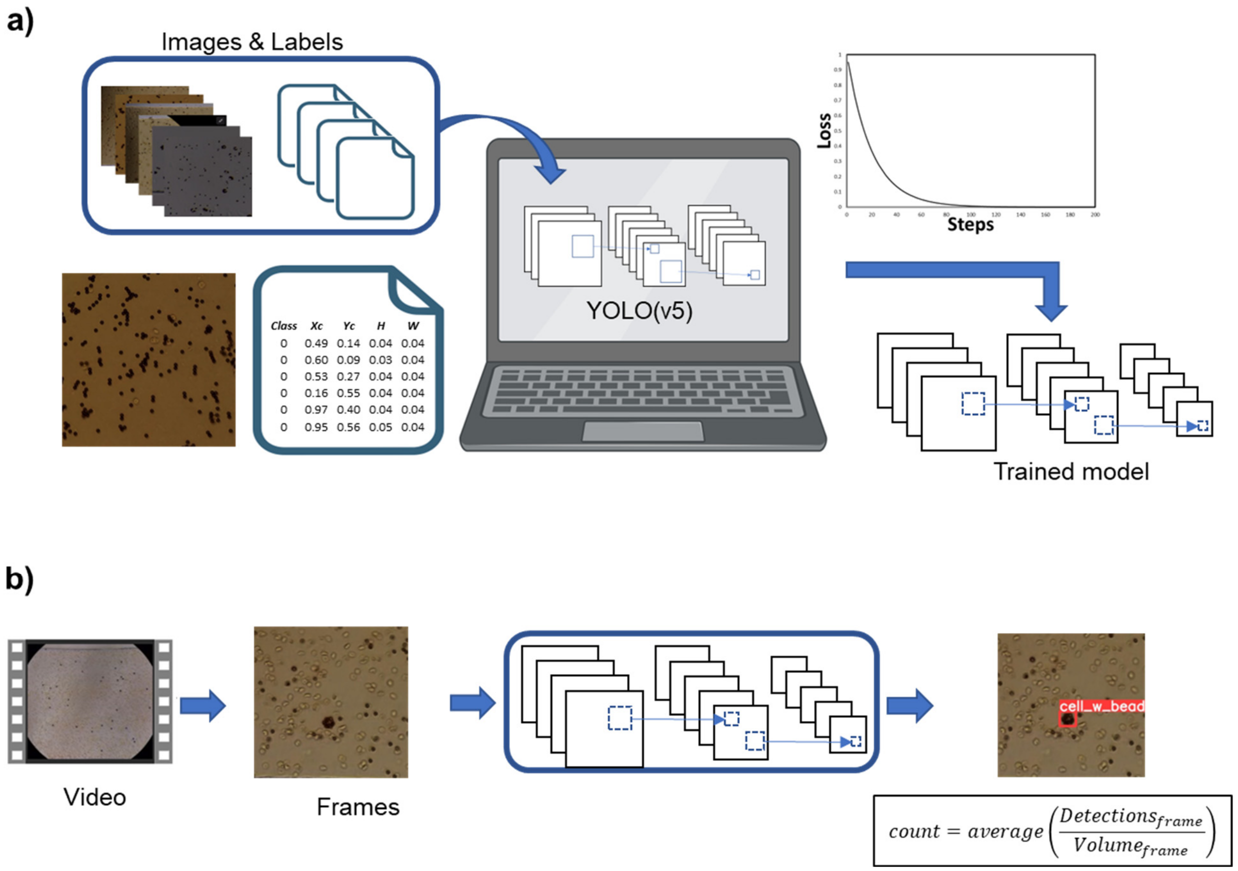

Fig. S6: Overview of the training and deployment of the ML algorithm. Schematic illustrations depicting (**a**) the ML algorithm training process, and (**b**) the deployment of the algorithm for $CD4^{+}$ T cell quantification in whole blood.

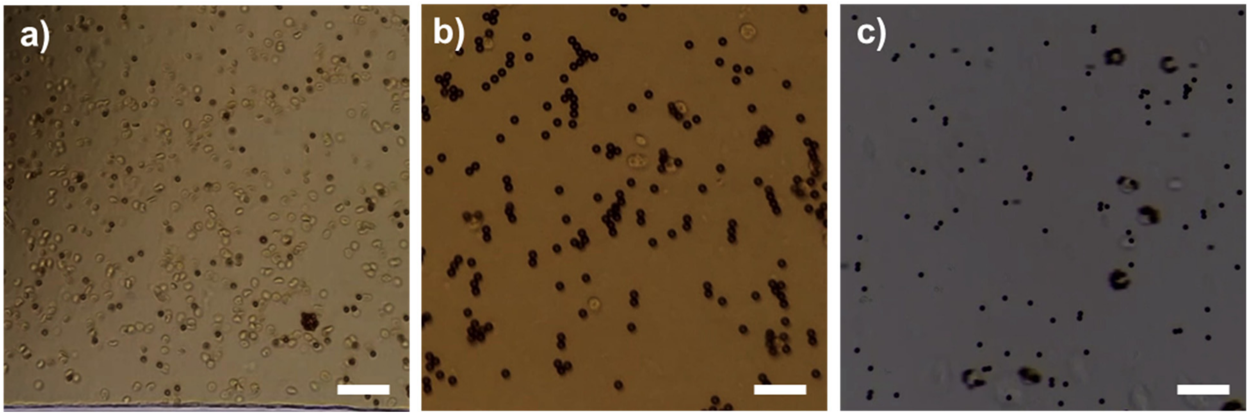

Fig. S7: Representative microscopy images used to train the ML model. Video frame images (640×640 pixels) of bead-labeled blood samples flowing through microfluidic chips with different (**a**) RBC concentrations, (**b**) bead concentrations and (**c**) lighting conditions. Scale bars, 50 µm.

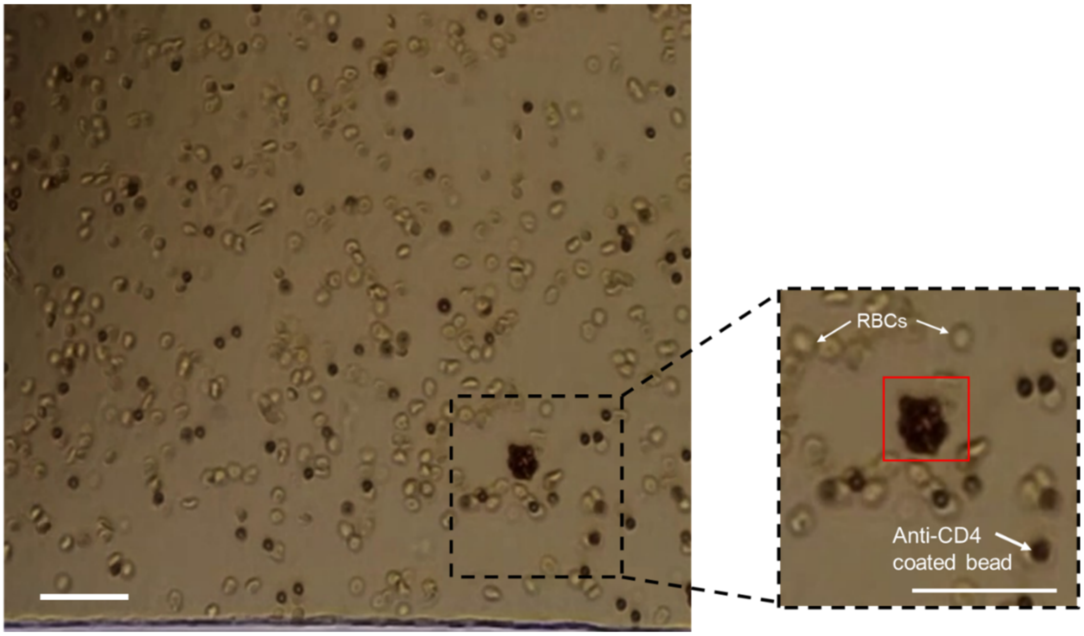

Fig. S8: Representative annotated microscopy image used to train the ML model. Video frame image (640×640 pixels) of a 10×-diluted blood sample with bead-labeled CD4^{+} T cells flowing through the microfluidic chip. Scale bar, 50 µm. Inset shows a magnified view of a bead-labeled CD4^{+} T cell outlined with a red box. Scale bar, 50 µm.

Table S1. Comparison of the AI-enabled microfluidic cytometer with conventional flow cytometry and impedance flow cytometry for $CD4^+$ T cell quantification.

Technique	Turnaround time[1]	Accuracy (absolute % error)	Equipment cost	Sample preparation	Throughput ($CD4^+$T cells/min)	Ref.
Flow cytometry	> 60 min	-	~$100,000	Extensive	~30,000	8–12
Impedance flow cytometry	20 min	~3%	~$15,000	Minimal	-	13
	10 min	-	~$15,000	Moderate	-	14
	5 min	~1.5%	$7,000	Minimal	-	15
AI-enabled microfluidic cytometer	15 min	~6%	~$1,000	Minimal	~7,333	This work

Notes:

[1] Includes the time needed for sample preparation and sample analysis

References

1. Kundu, P. K., Cohen, I. M. & Dowling, D. R. *Fluid mechanics*. (Academic press, 2015).
2. Bruus, H. *Theoretical microfluidics*. vol. 18 (Oxford university press, 2007).
3. Brown, P. H., Balbo, A., Zhao, H., Ebel, C. & Schuck, P. Density contrast sedimentation velocity for the determination of protein partial-specific volumes. *PLoS One* **6**, (2011).
4. Trudnowski, R. J. & Rico, R. C. Specific gravity of blood and plasma at 4 and 37 °C. *Clin. Chem.* **20**, 615–616 (1974).
5. Papaioannou, T. G., Stefanadis, C. & others. Vascular wall shear stress: basic principles and methods. *Hell. J Cardiol* **46**, 9–15 (2005).
6. Blachechen, L. S., Silva, J. O., Barbosa, L. R. S., Itri, R. & Petri, D. F. S. Hofmeister effects on the colloidal stability of poly(ethylene glycol)-decorated nanoparticles. *Colloid Polym. Sci.* **290**, 1537–1546 (2012).
7. Hrnčíř, E. & Rosina, J. Surface tension of blood. *Physiol. Res.* **46**, 319–321 (1997).
8. Nicholson, J. K. A., Jones, B. M. & Hubbard, M. CD4 T-lymphocyte determinations on whole blood specimens using a single-tube three-color assay. *Cytometry* **14**, 685–689 (1993).
9. Guidelines for the performance of CD4+ T-cell determinations in persons with human immunodeficiency virus infection. *MMWR Recomm. Reports* (1992) doi:10.1037/E546652006-001.
10. Imade, G. E. *et al.* Comparison of a New, Affordable Flow Cytometric Method and the Manual Magnetic Bead Technique for CD4 T-Lymphocyte Counting in a Northern Nigerian Setting. *Clin. Vaccine Immunol.* **12**, 224–227 (2005).
11. Secko, D. Inexpensive CD4 counting for the developing world. *CMAJ* **173**, 478 (2005).
12. Kala, P. S. & Zubair, M. Flow Cytometry Blood Cell Identification. in *StatPearls [Internet]* (StatPearls Publishing, 2024).
13. Watkins, N. N. *et al.* Microfluidic CD4+ and CD8+ T lymphocyte counters for point-of-care HIV diagnostics using whole blood. *Sci. Transl. Med.* **5**, (2013).
14. Watkins, N. N. *et al.* A microfabricated electrical differential counter for the selective enumeration of CD4+ T lymphocytes. *Lab Chip* **11**, 1437–1447 (2011).
15. Sher, M. & Asghar, W. Development of a multiplex fully automated assay for rapid quantification of CD4+ T cells from whole blood. *Biosens. Bioelectron.* **142**, 111490 (2019).
